# Increase in Male Reproductive Success and Female Reproductive Investment in Invasive Populations of the Harlequin Ladybird *Harmonia axyridis*

**DOI:** 10.1371/journal.pone.0077083

**Published:** 2013-10-18

**Authors:** Guillaume J. M. Laugier, Gilles Le Moguédec, Ashraf Tayeh, Anne Loiseau, Naoya Osawa, Arnaud Estoup, Benoît Facon

**Affiliations:** 1 Inra, Centre de Biologie pour la Gestion des Populations, Montpellier, France; 2 Inra, botAnique et bioinforMatique de l'Architecture des Plantes, Montpellier, France; 3 Laboratory of Forest Ecology, Graduate School of Agriculture, Kyoto University, Kyoto, Japan; CNRS, Université de Bourgogne, France

## Abstract

Reproductive strategy affects population dynamics and genetic parameters that can, in turn, affect evolutionary processes during the course of biological invasion. Life-history traits associated with reproductive strategy are therefore potentially good candidates for rapid evolutionary shifts during invasions. In a series of mating trials, we examined mixed groups of four males from invasive and native populations of the harlequin ladybird *Harmonia axyridis* mating freely during 48 hours with one female of either type. We recorded the identity of the first male to copulate and after the 48 h-period, we examined female fecundity and share of paternity, using molecular markers. We found that invasive populations have a different profile of male and female reproductive output. Males from invasive populations are more likely to mate first and gain a higher proportion of offspring with both invasive and native females. Females from invasive populations reproduce sooner, lay more eggs, and have offspring sired by a larger number of fathers than females from native populations. We found no evidence of direct inbreeding avoidance behaviour in both invasive and native females. This study highlights the importance of investigating evolutionary changes in reproductive strategy and associated traits during biological invasions.

## Introduction

Evolutionary processes and genetic attributes of invasive populations may underpin their success in becoming established in a new range [1]–[3]. Life-history traits associated with reproductive strategy are potentially good candidates for rapid evolutionary shifts during invasions [4], because reproductive strategy affects population dynamics and genetic parameters that can, in turn, have feedback effects on evolutionary processes [5]–[7]. Indeed, when species shift their range, they encounter a suite of new selective pressures that may affect their reproductive strategy. For instance, the lower population density at an expanding front would be expected to select for higher fecundity, lower age at first reproduction, or even a switch from outcrossing to selfing, all of which increase the individual's rate of reproduction [6], [8], [9]. Rapid evolution towards higher levels of reproduction following invasions may also result from a relaxation of selection for defence against enemies in the invaded range [10], [11]. In accordance with these expectations, a number of studies have shown that invasive populations can display increased reproductive efforts, have higher levels of reproductive investment, shorter generation times or higher selfing rates than native populations [6], [10], [12]–[17]. Reproductive strategy can also influence the adaptive potential of invasive populations. The purging of deleterious alleles and admixture between populations are crucial determinants of the fate of some invasions [18], [19]. In particular, low effective population sizes following the introduction should increase the proportion of mating between relatives and thus decrease the mean fitness of the population through inbreeding depression [20]. The response of a species to purging and admixture depends on its mating regime. For example, high selfing rates may slow admixture and accelerate purging, whereas multiple mating and allogamy would be expected to have the opposite effect.

Our current understanding of evolutionary shifts in the reproductive strategies associated with invasions is based largely on plant species (e.g. [6], [21], [22]). Moreover, most studies have dealt exclusively with female function (with no measurement of male function), focusing particularly on female reproductive effort [12], [23], [24]. Consequently, very little is known about the effects of invasion processes on other aspects of reproductive systems, including behavioural components, such as male-male competition for access to females and sperm competition. In particular, multiple mating (also referred to as promiscuous mating), although taxonomically widespread, has never been investigated in this context. Multiple mating is known to trigger the rapid evolution of sexual traits [25]. It can provide females with many advantages, such as ensuring fertilisation [26], the laying of larger numbers of eggs [27], greater genetic diversity of the progeny [28], [29] and sperm quality selection [30]. Multiple mating also has major evolutionary consequences for males [31], because the net reproductive success of an individual male is determined by his success in acquiring mates and copulating (*i.e.*, mating success), and by the number of eggs fertilised at each mating (*i.e.*, fertilisation success). Multiple mating may also incur considerable fitness costs, due to greater exposure to sexually transmitted disease, predation, a decrease in lifespan or the risk of physical harm to the female during copulation [32], [33]. In species displaying multiple mating, the reproductive success of both sexes depends on processes occurring both before (pre-mating) and after (post-mating) copulation [34].

In this study, we investigated the effects of invasion on reproductive traits in a species with multiple mating as a major reproductive strategy: the invasive harlequin ladybird *Harmonia axyridis* Pallas. *H. axyridis* is native to Asia and was introduced into North America and Europe as a biological control agent. It subsequently became invasive and has spread rapidly worldwide, with a complex invasion history involving admixture events in particular [35], [36]. *H. axyridis* displays multiple mating [37], the storage by females of sperm from multiple males [38], [39] and the production of up to three generations per year [40]. Studies comparing *H. axyridis* populations have indicated that this species has undergone rapid evolution during the invasion process. Firstly, invasive females have been shown to reproduce earlier than native females [41]. Secondly, while native populations display inbreeding depression, invasive populations do not, probably due to a purging process during invasion [41]. As inbreeding depression exerts a major selective pressure on the mating system, different reproductive traits may evolve in invasive populations in this species. However, it remains unclear whether any other aspects of mating behaviour differ between invasive and native populations.

The aim of this study was to clarify this point by investigating differences in reproductive success, for both males and females, between native and invasive populations of *H. axyridis*. We specifically aimed to determine whether (1) invasive males were at an advantage, in terms of both mating success (probability of being the first male to copulate) and fertilisation success (number of offspring sired), (2) invasive females copulated with more males, were more fecund and began to reproduce at younger age than native females, (3) there was a potential interaction between male and female origin (invasive vs. native) for these traits, and (4) whether there was a difference in inbreeding avoidance between native and invasive females.

## Materials and Methods

### Samples of *H. axyridis*

We used H. axyridis individuals sampled from five populations, subsequently reared in laboratory conditions for three generations to minimise maternal effects in mating experiments. Two of these populations came from the native area and were sampled from Beijing (China) and Fuchu (Japan). The other three populations came from the invaded area, and were sampled in Quebec City (Canada), Bataszek (Hungary) and Bethlehem (South Africa). The sampling was conducted in public locations that did neither require specific authorisation nor involve endangered or protected species. The samples included at least 50 individuals of each sex per population. All individuals were reared at 23°C, with 14∶10 L∶D and fed with an excess of ionised *Ephestia khueniella* eggs. We used pieces of black cardboard, folded above the food, as oviposition medium.

### Mating trials

We investigated mixed groups of four males from invasive and native populations mating with one female of either type, involving a total of 129 virgin females and four times as many males (See Table S1 in the supporting information). Females were set individually in arenas (Petri dishes, 7 cm in diameter) into which we had placed four virgin males the day before (see Figure S1 in the supplementary materials for details). Setting the females into the arena after the males ensured that the arenas were not saturated with females pheromones at the start of the experiment. The choice of the four males was inspired by a previous study [41], [42]. One of the males was a full-sib of the female, another was an unrelated male from the same population, and the remaining two males were from other populations, one native and the other invasive. The males were identified by means of coloured dots painted onto the elytra. The colour code was randomised to prevent confounding effects of marking. All individuals had emerged about 18 days before the experiment and had been kept alone, ensuring that they were all of the same age, sexually mature, but virgin [43], [44].

Once all five individuals were present in the arena, we observed them for one hour and recorded the identity of the first male to engage in copulation (successful mounting, phase iv as described by Obata [45]) with the female. The boxes were then left for 48 hours, during which time the individuals were allowed to copulate freely, and the males were then removed and preserved in ethanol for subsequent molecular analysis. Our experimental design differs from mating trials involving the sequential mating of a female with two different males, the second male being proposed to the female after the end of copulation with the first male [46]. The design of our experiment did not allow such a high level of control, and we only recorded the first mating. However, it better reflects natural conditions for a promiscuous species with multiple male partners available at the same time. It did allow free mating between a female and four males over a 48-hour period, including the possibilities of all males remating, not mating at all and possible mate guarding behaviour [47]. The first male to mate may, therefore, also have been the last. It has to be noted that our paternity analyses confirm that *H. axyridis* displays multiple mating [37] and the storage by females of sperm from multiple males. Indeed, the 48-hour period is enough for multiple mating to occur and most females (75%) in all populations studied mothered offspring from several males (see Results).

### Female reproductive investment

Female fecundity was assessed by checking the females for eggs every two to three days after the start of the experiment, and for 23–24 days following their first clutch. Similarly to a previous work [41], we followed the females for up to 63 days (corresponding to the time required for 80% of the females to lay eggs). The remaining females were assumed to be sterile or to have not mated successfully. We recorded the date of the first clutch and the total number of eggs laid during this period. Once counted, the egg clutches were transferred to individual Petri dishes and fresh oviposition medium was provided to the females. The hatching rate was estimated for a mean of three clutches per female. Two of these three clutches per female, laid at least 10 days apart, were allowed to develop to the second larval stage, which was stored in ethanol for subsequent molecular analysis.

### Male reproductive success

Male mating success was estimated by the identity of the male engaged in the first mating. Male fertilisation success was evaluated by the number of offspring sired by each of the four males for all studied females. We genotyped microsatellite loci in up to eight larvae per clutch for the two clutches per female allowed to develop to the second larval stage. We also genotyped all males and females. We used the eight most variable microsatelite loci of those described by Loiseau et al. [48]. Paternity was assessed with PROBMAX software [49]. We found no difference in paternity pattern between the early and late clutches, which were therefore pooled for data analysis. Coupling percentage paternity with the results of the mating trials allowed distinction between mating success and fertilisation. It also allowed the comparison of the realized paternity between different males while controlling for first sperm precedence. The effective number of fathers per female was calculated as *E_f_ = 1/∑_i_ f_i_^2^*, where *f_i_* is the frequency of paternity for male *i*.

### Modelling and statistical analyses

We used classic parametric (*t*-test, Binomial GLM) and non-parametric (Kruskal-Wallis rank-sum test) statistical tests to assess statistical differences in female traits. We recorded the percentage of egg-laying females, the daily fecundity of egg-laying females and the hatching rate. These traits were analysed with respect to the female origin (native vs. invasive) and the characteristics of the first male copulating with the female or siring the majority of the female's offspring. The male characteristics considered were origin and being a full sibling of the female concerned.

We studied male reproductive success by calculating the probability of a male being the first to copulate, and its percentage paternity among the offspring of the female as a function of his characteristics and those of the female. The male characteristics considered were origin (native vs. invasive), population, the relatedness to the female (whether or not the male and the female were full siblings) and, when applicable, involvement in the first copulation. The female characteristics studied were origin (native vs. invasive) and population. We also investigated the potential effects of the body size (measured as the length of elytron) and colour morph of both sexes in preliminary statistical treatments. These two factors were found to have no significant effect (results not shown) and were, therefore, not included in the factors considered in the statistical models presented below.

As the explanatory variables were potentially subject to complex interactions or nesting, we studied the response variable (*i.e.* probability of first copulation and percentage paternity) by a modelling approach. Males were in competition with each other within each Petri dish, and the reproductive success of a given male depended not only on his own characteristics, but also on those of his competitors. The non-independence of the reproductive success of the individual males in each Petri dish precluded the use of classical generalised linear models. The competition between males in terms of copulation and percentage paternity is essentially like a race between competitors. We therefore use multinomial models classically used in competition analysis (e.g. [50], [51]). Our modelling approach is described in detail in the supplementary materials. In brief, the probability of a given male copulating with the female or being the father of the offspring in a particular egg is a function of the characteristics of this focal male with respect to those of the other males present in the arena. In each round, four males ‘run to the finish line’ (mounting the female), so the probability 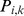 of a male *k* winning a race in ‘arena’ *i* (a given Petri dish) depends on its ‘fitness score’ 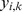 relative to the other three males.

This probability can be written
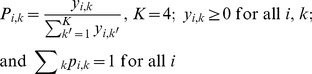
where the score 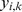 can be generally written as
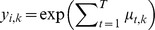
with 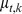 the effect of factor 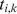 (including interaction factors) for male *k* in arena *i*.

Starting with a null model in which each male has the same probability of success, we then added the effects (or interactions of effects) to be tested. Comparing nested models with likelihood ratio-tests allowed testing if each effect or interaction of effects improves the model [52]. For the relevant models, confidence intervals were calculated with bootstraps of 2,000 samplings and used to test separately the effects that would otherwise been confounded.

The R software [53] was used for both the classical statistical analysis and the modelling approach.

## Results

### Male reproductive success

During the first hour of the experiment, 93% of females engaged in copulation (see Table S1, and Dataset S1 in the supporting information for details). We analysed the traits of the males only for these females.

#### Invasive males tend to be the first to copulate

**Figure 1:**
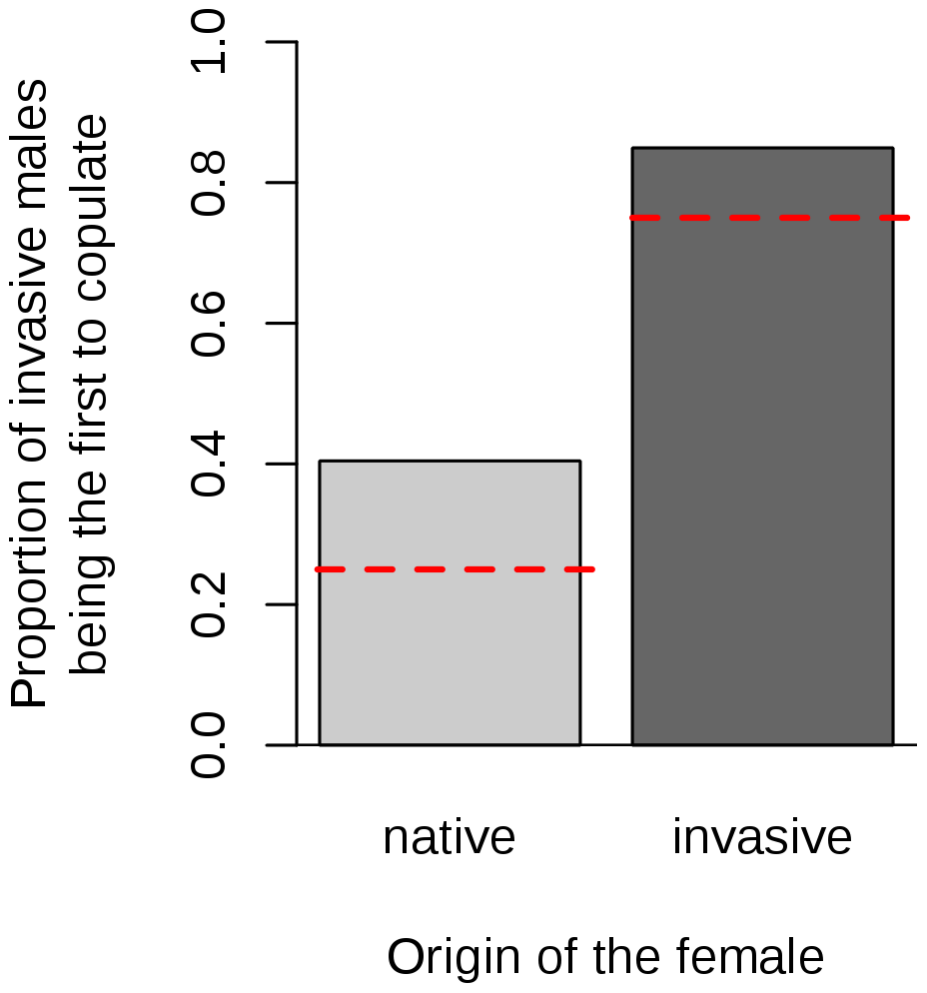
Effect of the origin of the male on his probability of being the first to copulate. Observed proportion of invasive males being the first to copulate with native and invasive females. The red dashed lines represent the proportion expected under the null hypothesis.

**Figure 2:**
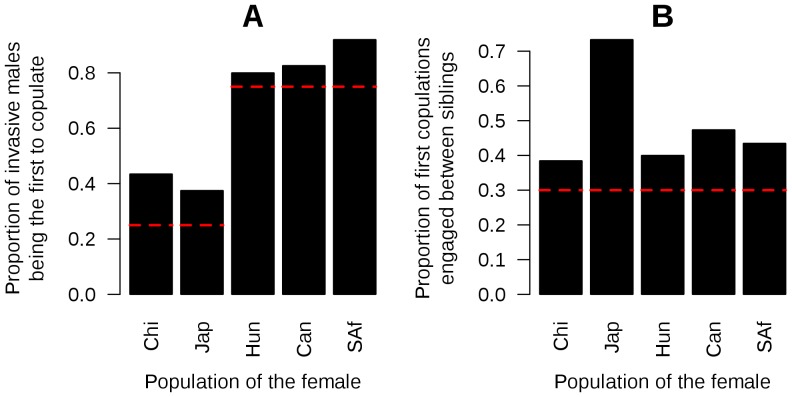
Proportion of females that engaging in a first copulation with an invasive male (A) or with their brother (B). The red dashed lines are the proportions expected under the null hypothesis (random mating). In panel B, the proportion is calculated for the females first copulated with a male of the same origin (native vs. invasive) only. Chi: China, Jap: Japan, Hun: Hungary, Can: Canada, SAf: South Africa.

In our experimental design, native females were presented with three native males and one invasive male each, so 25% of first copulations would be expected to be with the invasive male if mate choice were random. Conversely, invasive females were presented with one native male and three invasive males, so 75% of first copulations would be expected to be with invasive males in a context of random choice. We found that 40% of native females and 85% of invasive females first copulated with an invasive male (Figure 1), regardless of the population of the male (see Table S1 in supporting information). Our modelling-based analysis indicated that being invasive had a significant effect on the model likelihood of being the first male to copulate (*P* = 1.9×10^−3^, Table 1, model C1). The estimated effect was positive and significantly different from zero (Table 2, model C1), and no significant difference in estimated effect was found between native and invasive females (*P*>0.1, Table 1, model C2) or between populations (Figure 2A; Table 1, models C1.2 and C2.2).

**Table 1:** Statistical models of probability of being the first male to copulate.

	Models	df	Log(L)	Test statistic*	P*
C0	null model	0	−166.4		
C1	♂ origin	1	−161.5	X^2^_1_ = 9.62	**1.9×10^−3^**
C1.2	♂ population	4	−160.5	X^2^_3_ = 2.05	0.56
C2	♂ origin: ♀ origin	2	−161.5	X^2^_1_ = 0.03	0.86
C2.2	♂ origin+♂ origin : ♀ population	5	−159.3	X^2^_3_ = 4.44	0.22
C3	♂ origin+sibling‡	2	−158.2	X^2^_1_ = 6.7	**9.6×10^−3^**
C4	♂ origin+sibling‡: origin	3	−157.7	X^2^_1_ = 0.94	0.33
C4.2	♂ origin+♂ sibling+sibling‡ : population	6	−156.2	X^2^_3_ = 6.32	**0.097**

* Test statistics and *P*-values from Chi-squared tests of the differences of log likelihood.

‡Whether or not the male was a full sibling of the female. Colons represent interaction factors, according to the conventions of the R language.

**Table 2:** Estimated effects in models of probability of being the first male to copulate.

Models	Estimate	P(x≠0)†
C1	x_invasive♂_ = 0.67	**
C3	x_brother_ = 0.51	*
	x_invasive♂_ = 0.70	**
C4.2	x_brother:japan_ = 1.11	-
	x_brother:hungary_ = −0.01	NS
	x_brother:canada_ = 0.37	NS
	x_brother:S.africa_ = 0.33	NS
	x_brother_ = 0.16	NS
	x_invasive♂_ = 0.80	*

†Significance code for probability of the effect estimate being different from zero (the effect taken as a reference), using a bootstrap of 2,000 replicates.

** *P*≤0.01;

* *P*≤0.05;

- *P*≤0.1;

NS *P*>0.1.

Colons represent interaction factors in accordance with the conventions of R language. Only relevant models are presented.

#### Invasive males have a higher percentage paternity

**Figure 3:**
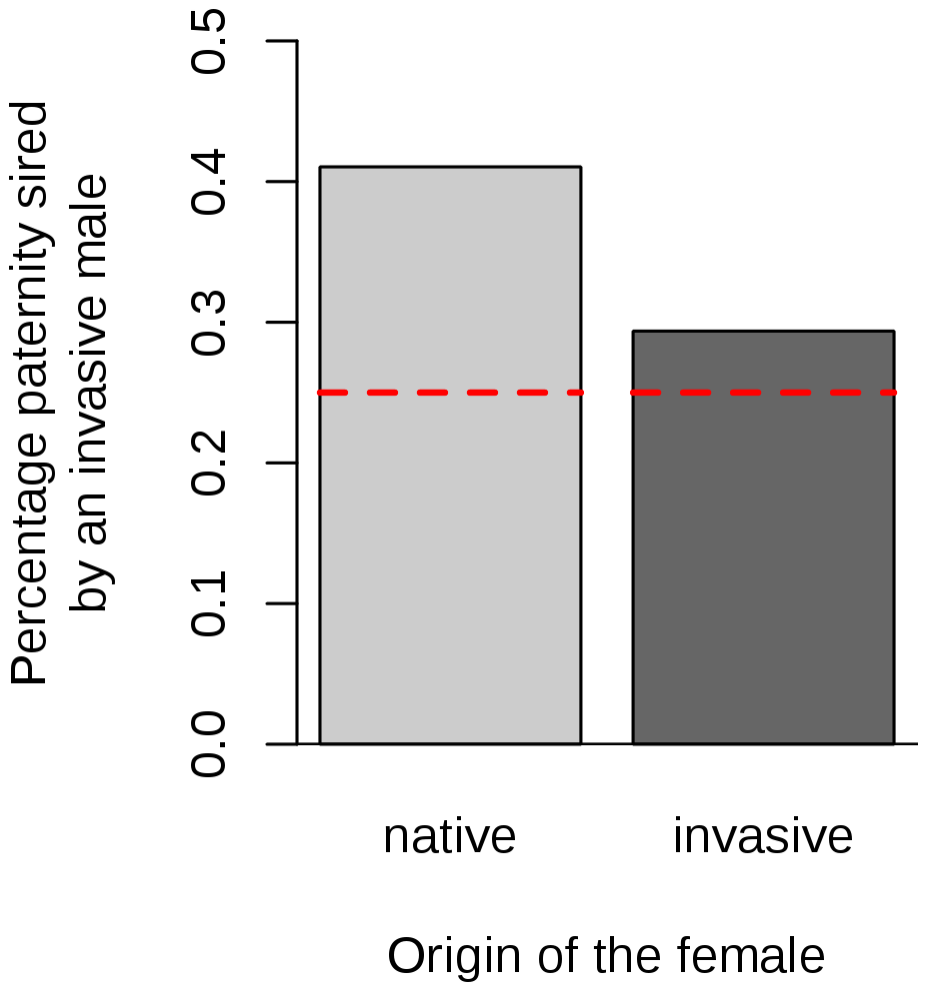
Effect of the origin of the male on his percentage paternity. Average observed percentage paternity of each invasive male copulating with a native or an invasive female.

According to the null model, each male should sire 25% of the female's offspring. However, we found that invasive males sired, on average, 41% of the offspring when mating with a native female, and 29% of the offspring when mating with an invasive female (Figure 3A). As invasive males are more likely to mate first, we used our statistical modelling approach to take the advantage of being the first male to mate into account (Table 3, model P1), then estimated the effect of being invasive on his percentage paternity among the offspring. The inclusion of an effect of being invasive significantly improved the model (*P* = 7.7×10^−16^, Table 3, model P3), and this effect was positive and significant (Table 4, model P3), although smaller than that of being the first male to copulate. Allowing different estimations between male populations significantly improved the model (Table 3, model P3.2). However, the pattern with two categories of males (Native vs. Invasive) was mainly found again, except that South African males were not significantly different from native ones (Table 4, model P3.3).

**Table 3:** *Statistical models* of percentage paternity.

	Models	df	Log(L)	Test statistic*	P*
P0	null model	0	−1255		
P1	first†	1	−990.1	X^2^_1_ = 530	**3.5×10^−117^**
P2	first†+♀ origin	2	−980.6	X^2^_1_ = 18.98	**1.3×10^−5^**
P2.2	first†+first† :♀ population	5	−886.1	X^2^_3_ = 140.94	**2.4×10^−30^**
P3	first†+♂ origin	2	−957.6	X^2^_1_ = 64.9	**7.7×10^−16^**
P3.2	first†+♂ population	5	−942.6	X^2^_3_ = 30.122	**1.3×10^−6^**
P4	first†+♂ origin: ♀ origin	3	−956.6	X^2^_1_ = 2.03	0.15
P4.2	first†+♂ origin+♂ origin : ♀ population	6	−886.1	X^2^_4_ = 143.0	**6.5×10^−30^**
P5	first†+♂ origin+sibling‡	3	−955.3	X^2^_1_ = 4.59	**0.032**
P6	first†+♂ origin+sibling‡: origin	4	−955.3	X^2^_1_ = 0.08	0.78
P6.2	first†+♂ origin+♂ origin+sibling‡: population	7	−892.5	X^2^_3_ = 125.5	**3.1×10^−27^**

* Test statistics and *P*-values from Chi-squared tests of the differences of log likelihood.

†Whether or not the male was the first to copulate with the female.

‡Whether or not the male was a full sibling of the female. Colons represent interaction factors, according to the conventions of the R language.

**Table 4:** Estimated effects in models of percentage paternity.

Models	Factor	Estimate	P(x≠0)†
P1	first copulation	1.29	**
P2	first:invasive♀	0.51	NS
	first copulation	1.17	**
P2.2	first:japanese♀	−0.28	NS
	first:hugarian♀	−0.69	NS
	first:canadian♀	−0.76	NS
	first:S.african♀	−0.60	NS
	first copulation	1.78	**
P3	first:invasive♂	0.59	**
	first copulation	1.24	**
P3.2	first copulation	1.23	**
	japanese♀	0.28	NS
	hungarian♀	0.98	**
	canadian♀	0.86	**
	S.african♀	0.46	NS
P4.2	invasive♂:japanese♀	−1.04	NS
	invasive♂:hungarian♀	−0.68	NS
	invasive♂:canadian♀	−0.43	NS
	invasive♂:S.african♀	0.56	NS
	invasive♂	0.99	-
	first copulation	1.23	*
P5	brother	−0.15	NS
	invasive♂	0.58	**
	first copulation	1.26	**
P6.2	brother♂:japanese♀	1.21	NS
	brother♂: hungarian ♀	1.09	NS
	brother ♂: canadian ♀	0.88	NS
	brother ♂: S.african ♀	0.51	NS
	brother	−0.92	NS
	invasive♂	0.57	**
	first copulation	1.25	**

†Significance code for probability of the effect estimate being different from zero (the effect taken as a reference), using a bootstrap of 2,000 replicates.

** *P*≤0.01;

* *P*≤0.05;

- *P*≤0.1;

NS *P*>0.1.

Colons represent interaction factors in accordance with the conventions of R language. Only relevant models are presented.

There was no significant difference in the effect of being an invasive male between native and invasive females (Table 3, model P4). Allowing the effect of being invasive to differ between female populations significantly improved the model, (Table 3, model P4.2) but the differences between populations were not significant (Table 4, model P4.2).

### Does inbreeding avoidance occur?

#### Females do not avoid copulation with their brothers

According to the null model 25% of the females should first copulate with their brother. We found that 34% of native females and 37% of the invasive females first copulated with their brother. Once the advantage of being invasive was taken into account by the model, being a full sibling was found to have a significant effect on the probability of being the first male to copulate (*P* = 9.6×10^−3^; Table 1, model C3). The estimated effect of being a full sibling was positive and significant (Table 2, model C3). The model was not improved by allowing differences between native and invasive females (Table 1, model C4).

The Japanese females tended to be more likely to mate with their brothers than were the females of other populations (Figure 2B; Tables 1 and 2, model C4.2). This trend was only marginally significant, but this study lacked statistical power for analyses at the population scale. However, if we fitted the model to the data without the Japanese population (equivalent to model C3 in Table 1), the effect of being a full sibling was not significant (data not shown).

#### Percentage paternity of brothers

According to the null model, each male would be expected to sire 25% of the female's offspring. We found that the brothers of the females sired, on average, 26±3% of the female's offspring, with each of the other three males siring, on average, 25±0.1% of the offspring. This direct result does not take into account the effects of being the first male to copulate and being invasive into account.

Including the effect of being the female's brother in model P3, in which these effects were already present, significantly improved the model (*P* = 0.032, Table 3, model P5). The estimated effect was negative, but was not significantly different from zero (*P*>0.1, Table 4, model P5), indicating a slight disadvantage of being the female's brother in terms of paternity success. The model was not improved by allowing the effect of being the female's brother to differ between native and invasive populations (Table 3, model P6), but the model was improved by allowing this effect to differ between populations. However, the differences between populations were not significant (Table 4, model P6.2).

### Female reproductive investment

#### Invasive females are more fecund and lay eggs earlier than native females

**Figure 4:**
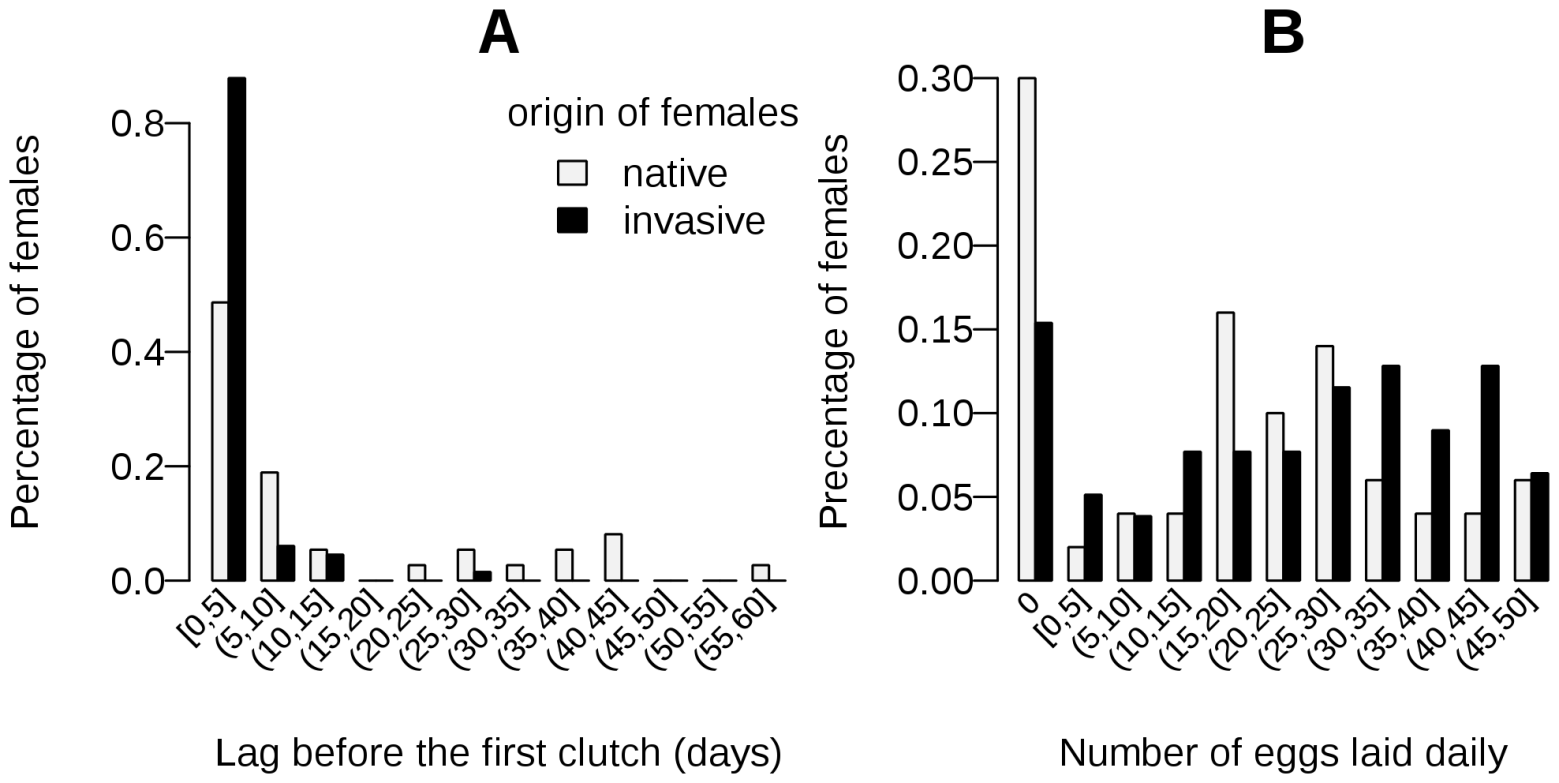
Fecundity traits of native and invasive females. **A:** Distribution of the time to first clutch after the presentation of native and invasive females to the males. The difference between the mean times to first clutch of native and invasive female is significant (Table 5). **B:** Distribution of the mean number of eggs per day laid by a native and by an invasive female. The means for native and invasive females are significantly different (see Table 5).

During the first hour of the experiment, 92% of native females and 95% of invasive females engaged in copulation. This difference is not significant (Fisher's exact test *P* = 0.71). Females that did not mate during the first hour laid significantly fewer eggs than those that did (*P* = 0.02, Table 5). Native females laid their first clutch 13±2.7 days after the start of the experiment, whereas invasive females started laying after only 2.2±0.5 days (Wilcoxon rank-sum test *P* = 6.43×10^−7^, Table 5; and Figure 4A). Native females laid fewer eggs than invasive females during the period studied (mean ± SEM = 17.7±2.1 and 22.7±1.6 eggs per day for native and invasive females, respectively). This difference is significant (Kruskal-Wallis rank-sum test *P* = 0.041, Table 5) and can be broken down into a trend towards higher proportions of egg-laying females within invasive populations and a trend for these females to lay more eggs per day (Figure 4B). These trends were not significant when considered separately (*p*>0.05; *Table* 5). We found no significant difference in hatching rate between native and invasive females (0.7±0.03 for both native and invasive females, Table 5). No significant difference was found between populations within status for any of these traits (data not shown).

**Table 5:** Statistical tests of the effects of female factors on female traits.

Effects	Response variable	Values (% or mean ± SE)	Type of test	Test statistic	Probability
Female factors					
♀ copulation*	♀ eggs laid daily (eggs/day)	copulation: 9.2±4.5; no copulation: 21.5±1.4	Kruskal-Wallis rank sum test	X^2^_1_ = 5.3	**0.0213**
♀ origin	daily fecundity (eggs/day)				
	- all ♀	native: 17.7±2.1; invasive: 22.7±1.6	Kruskal-Wallis rank sum test	X^2^_1_ = 4.170	**0.0415**
	- only egg-laying ♀	native: 24.4±2; invasive: 26.4±1.5	F test	F = 0.70	0.4042
♀ origin	% of egg-laying ♀	native: 72.5%; invasive: 85.7%	Likelihood Ratio Test	X^2^_1_ = 3.32	0.070
	♀ mean hatching rate	native: 0.673±0.034; invasive: 0.657±0.027	Kruskal-Wallis rank sum test	X^2^_1_ = 6.04	0.6628
	Time to first clutch (days)	native: 13.3±2.7; invasive: 2.2±0.5	Kruskal-Wallis rank sum test	X^2^_1_ = 24.8	**6.32×10^−7^**

* Whether or not copulation occurred during the first hour of the experiment.

#### Multiple paternity of offspring

Most of the females producing larvae that survived to the second larval stage (75%) mothered offspring from two to four different males. Invasive females were fertilised by a significantly larger number of fathers (2.18±0.13 an mean ± SEM = 1.62±0.14 fathers for invasive and native females, respectively; Kruskal-Wallis rank sum test: *X^2^_1_* = 9.48, *P* = 2.07×10^−3^). We found no significant difference between native populations. The Hungarian females mated with slightly more individuals than other invasive ones, but the difference was only marginally significant (Kruskal-Wallis rank sum test: *X^2^_2_* = 5.95, *P* = 0.05). Invasive females also were fertilised by a higher effective number of fathers than native females (1.68±0.10 and mean ± SEM = 1.32±0.09 effective fathers for invasive and native females, respectively; Kruskal-Wallis rank sum test: *X^2^_1_* = 8.58, *P* = 3.4×10^−3^). We found no significant difference between invasive populations. The Japanese females had offspring from slightly more effective fathers than Chinese ones, but the difference was only marginally significant (Kruskal-Wallis rank sum test: *X^2^_1_* = 3.93, *P* = 0.05).

#### Influence of males on female fecundity

Among females laying viable eggs, fecundity was higher if the principal father was a full sibling, for invasive females (mean ± SEM = 30.0±2.2 eggs per day for females whose brother sired most offspring, and 18.0±1.5 for other females, *P* = 3.6×10^−3^, Table 6), but not for native females (mean ± SEM = 22.3±2.9 eggs per day for brothers, and 15.9±2.3 for other males, *P*>0.1; Table 6). Although significant in South African and Hungarian females, this differences was not significant for the Canadian female (*P*>0.1). The origin (invasive/native) of the first male to copulate or the principal father of the offspring had no effect on any other female trait (male factors in Table 6).

**Table 6:** Statistical tests of the effects of male factors on female traits.

Effects	Response variable	Values (% or mean ± SE)	Type of test	Test statistic	Probability
first† ♂ origin	% of egg-laying ♀	native: 93%; invasive: 97%	Pearson's Chi-squared test	X^2^_1_ = 0.084	0.777
major‡ ♂ origin	♀ daily fecundity (eggs/day)	native: 25.0±1.9; invasive: 25.3±1.6	Kruskal-Wallis rank sum test	X^2^_1_ = 0.043	0.835
	♀ mean hatching rate	native: 0.69±0.03; invasive: 0.69±0.02	Kruskal-Wallis rank sum test	W = 885	0.929
first‡ ♂ sibling	% of egg-laying ♀	brother: 86%; other: 81%	Pearson's Chi-squared test	X^2^_1_ = 2.297	0.586
major† ♂ sibling	♀ daily fecundity (eggs/day)				
	- all ♀	brother: 30.0±2.2; other: 18.0±1.5	Kruskal-Wallis rank sum test	X^2^_1_ = 11.6	**6.7×10^−4^**
	- native ♀	brother: 22.3±2.9; other: 15.9±2.3	Kruskal-Wallis rank sum test	X^2^_1_ = 1.6	0.21
	- invasive ♀	brother: 32.8±2.5; other: 19.6±1.9	Kruskal-Wallis rank sum test	X^2^_1_ = 9.83	**1.7×10^−3^**
	♀ mean hatching rate	brother: 0.64±0.04; other: 0.67±0.02	Kruskal-Wallis rank sum test	X^2^_1_ = 1.2	0.27

†First male to copulate with the female.

‡Male siring most of the female's offspring.

## Discussion

Our results show that invasive populations display changes in both male and female traits associated with reproductive strategy, whose are expected to be selected for during a biological invasion [4], [54].

One of the key results of this study is that invasive males have a higher probability of being the first to copulate with both native and invasive females. This result indicates the absence of assortative mating [55] with respect to native/invasive origin and population. The advantage of invasive males over native ones may reflect a more active reproductive behaviour, a better ability to detect and locate females or a greater locomotive ability [56]–[58]. Indeed, despite of the small size of the arenas, many females ran away from males for a few minutes before accepting copulation. Hence, males with greater locomotory behaviour had more chance to be the first to detect the female than males that stayed still.

Early in the invasion process (and continually at the invasion front) population density is low, reducing the probability of mate encounters (an aspect of the Allee effect, see Elam et al. [59], for example). Higher levels of male sexual activity increase the chances of finding and mating with a female, and are therefore expected to be selected for during invasions [60].

We also found that invasive males have the advantage of siring a greater percentage of the offspring. Moreover, although we found a strong sperm precedence for the first male to copulate (Tables 3 and 4 of this article and figure S2 in supporting information), invasive males sired a greater proportion of the offspring than native ones, even if they were not the first to copulate. This suggests that invasive males outperform native males in terms of both sperm defence and offence [61], [62] This feature might come from invasive males producing higher quality, more competitive sperm [63], or larger ejaculates that might dilute or displace the sperm of previous males [46], [64]. Alternatively, females might exert directional post-copulation sexual selection in favour of invasive males [25]. Wang Su et al. [65] previously suggested that *H. axyridis* might display cryptic female choice. Unfortunately, our experiments do not allow testing any of these (non-exclusive) hypotheses and further experiments are needed to do so. Regardless of the mechanisms involved, our results are consistent with selection for more sexually competitive males as expected during the invasion process, because the populations densities, although low in the early stages of an invasion can be very high at the outbreak in later stages [66].

This pattern of higher percentage paternity was found for all invasive males, even though it was less pronounced in South African males. Although all these populations are genetically very close (data not shown), a certain amount of variation between invasive populations was indeed expected, because all the populations used in this study have a different invasion scenario [36], and thus possibly different density histories. Unfortunately, information on densities in these populations is scarce if any. Moreover, with only 2–3 populations of each type, the experiment was not designed to test any effects at population level, and such result should be interpreted with caution.

Native and invasive populations also differed in terms of female reproductive traits. This result provides an additional evidence, monitored over a longer period of time, for greater fecundity, beginning earlier in invasive females of *H. axyridis* (see Facon et al. [41] for initial evidence of this). At least in the early stages, invasive populations are typically in a state of demographic disequilibrium, with little or no regulation by density [67]. This demographic setting may result in the selection for higher levels of fecundity and on earlier onset of reproduction, both of which would accelerate population growth [8].

Invasive females were also fertilised by both a higher total number of fathers and a higher effective number of fathers. This may be the result of a post-copulation selective mechanism. Note that this result may also be at least partly due to our experimental design in which invasive females were presented with one native male and three invasive (therefore more competitive) males, whereas native females were presented with three native males and one invasive male. A higher number of effective fathers would ensure greater genetic diversity in the offspring of every single female [68]. This kind of bet-hedging strategy might make admixture more efficient and could be a key element of adaptation to new environments [68], [69], especially in the context of a biological invasion, where genetic diversity that may have decreased during the introduction process could be restored [18], [70].

Finally, we were interested in determining whether native and invasive individuals displayed different levels of inbreeding avoidance. Facon et al. [41] found that native populations of *H. axyridis* displayed inbreeding depression, whereas invasive populations did not, probably due to the occurrence of a purging process during invasion. Although we expected direct inbreeding avoidance behaviour to occur, at least in the native populations, this study provided no evidence of such mechanism in *H. axyridis*. On the contrary, we found that the brothers of the females tested had a slight, but significant advantage over the other males, increasing the likelihood of copulating first with their sisters, in both native and invasive populations. This advantage might only genuinely exist in the Japanese population studied, but we cannot tell if it reflects a particular feature of the Japanese population as we used only two native populations. Regardless, we found no trend for individuals to avoid copulation with siblings in any population, and the weak negative impact of kinship on paternity did not seem to be strong enough to be considered as an actual direct mechanism of inbreeding avoidance. The probability of encounter of a sibling may be low in the field, for instance, if dispersal occurs before sexual maturity [71], [72] as it occurs in *H. axyridis* [73]. This might explain why *H. axyridis* individuals have no mechanisms for preventing copulation between full siblings in the conditions of our experiment. Besides invasive females are, on average, more fecund when fertilised with their brothers contrary to native ones. This result could be linked with the absence of the cost imposed by inbreeding depression in invasive populations [73], and might be explained by kin selection in a context of mate limitation such as expected in the first steps of invasion or at the invasion front.

In conclusion, reproductive traits are expected to evolve during the invasion process as a result of changes in population densities and selective pressures. Our study shows that invasive populations of *Harmonia axyridis* display higher levels of reproductive investment in both males and females. Interestingly, we found no major interaction between male and female origin on the probability of copulation or its outcome. Invasive males and females have thus higher reproductive success regardless of the origin of their partner. This result matches well the theoretical expectations in this particular evolutionary context [4], [54]. Contrary to expectations [41] we found no evidence of inbreeding behaviour during pre- nor post-copulation competition processes in native or invasive populations, suggesting that another mechanism would exist at least in native populations [74]. More work is needed to test this assumption. We hope that this study will stimulate further research into the evolution of reproductive strategy and associated traits during invasion processes.

## Supporting Information

Table S1 Summary table of the copulation results. * Population of origin: Chi: China, Jap: Japan, Hun: Hungary, Can: Canada, SAf: South Africa. † Number of females that laid eggs. ‡ Number of which copulated with their brother first. ¶ Number of females with viable eggs. § Number of females with genotyped larvae. The last three columns only include females that copulated during the first hour, i.e. for which first copulation data were available.
(DOCX)

Figure S1 **Design of the mate choice experiment.** Each female was placed in a Petri dish containing four males: a full sibling, another unrelated male from the same population and two males from other populations, one native and one invasive. The identity of the first male to mount the female was recorded during the first hour and the insects were then left to copulate freely for the next 47 hours. The males were then removed and the females were left alone for 23 days after the laying of the first clutch of eggs. During this period, female fecundity was recorded and the hatching rate of the eggs was estimated from at least one early and one late clutch. The paternity of eight second-instar larvae was assessed by molecular analysis, in two clutches laid at least 10 days apart.
(TIFF)

Figure S2 **Effect of being the first male to copulate with a given female on percentage of paternity within the female's offspring.** **A**: Observed percentage paternity of the first male to copulate with native and invasive females. Red dashed lines are the values expected under the null hypothesis. **B**: Model estimates of the effect of being the first male to copulate with a female on percentage paternity, with corresponding 95% confidence intervals. The expected effect with the null model is zero for both native and invasive females. The effect is significant in both cases, but the difference between native and invasive females is not significant (see models P1 and P2 in Tables 2 and 4).
(TIFF)

Dataset S1 **Experimental data used in this study.** This spreadsheet contains the results of the copulation experiments as well as the paternity assignment obtained from microsatellite genotypes.
(ODS)

Appendix S1 **Details of the statistical model of competition.**
(DOCX)
