# Supplementary material for: Increase in Male Reproductive Success and Female Reproductive Investment in Invasive Populations of the Harlequin Ladybird Harmonia axyridis
Source: PLoS One. 2013 Oct 18;8(10):e77083. doi: 10.1371/journal.pone.0077083 (PMC3799855; doi:10.1371/journal.pone.0077083)
Supplement: Table S1 — Summary table of the copulation results. * Population of origin: Chi: China, Jap: Japan, Hun: Hungary, Can: Canada, SAf: South Africa. † Number of females that laid eggs. ‡ Number of which copulated with their brother first. ¶ Number of females with viable eggs. § Number of females with genotyped larvae. The last three columns only include females that copulated during the first hour, i.e. for which first copulation data were available. (DOCX) [file pone.0077083.s001.docx]

| Population* of  the female | Population* of the first male to copulate | | | | | Eggs^†^ | Hatch^¶^ | Larva^§^ |
| --- | --- | --- | --- | --- | --- | --- | --- | --- |
|  | Chi | Jap | Hun | Can | SAf |  |  |  |
| Chi (26) | 10 (5^‡^) | 3 | 3 | 3 | 4 | 18 | 17 | 17 |
| Jap(25) | 2 | 13 (11^‡^) | 3 | 4 | 2 | 18 | 18 | 18 |
| Hun (26) | 3 | 2 | 15 (8^‡^) | 4 | 1 | 22 | 21 | 21 |
| Can (26) | 2 | 2 | 4 | 14 (9^‡^) | 1 | 22 | 21 | 20 |
| SAf (26) | 0 | 2 | 7 | 2 | 14 (10^‡^) | 22 | 20 | 20 |
