# Supplementary figures and images for: Increase in Male Reproductive Success and Female Reproductive Investment in Invasive Populations of the Harlequin Ladybird Harmonia axyridis
Source: PLoS One. 2013 Oct 18;8(10):e77083. doi: 10.1371/journal.pone.0077083 (PMC3799855; doi:10.1371/journal.pone.0077083)

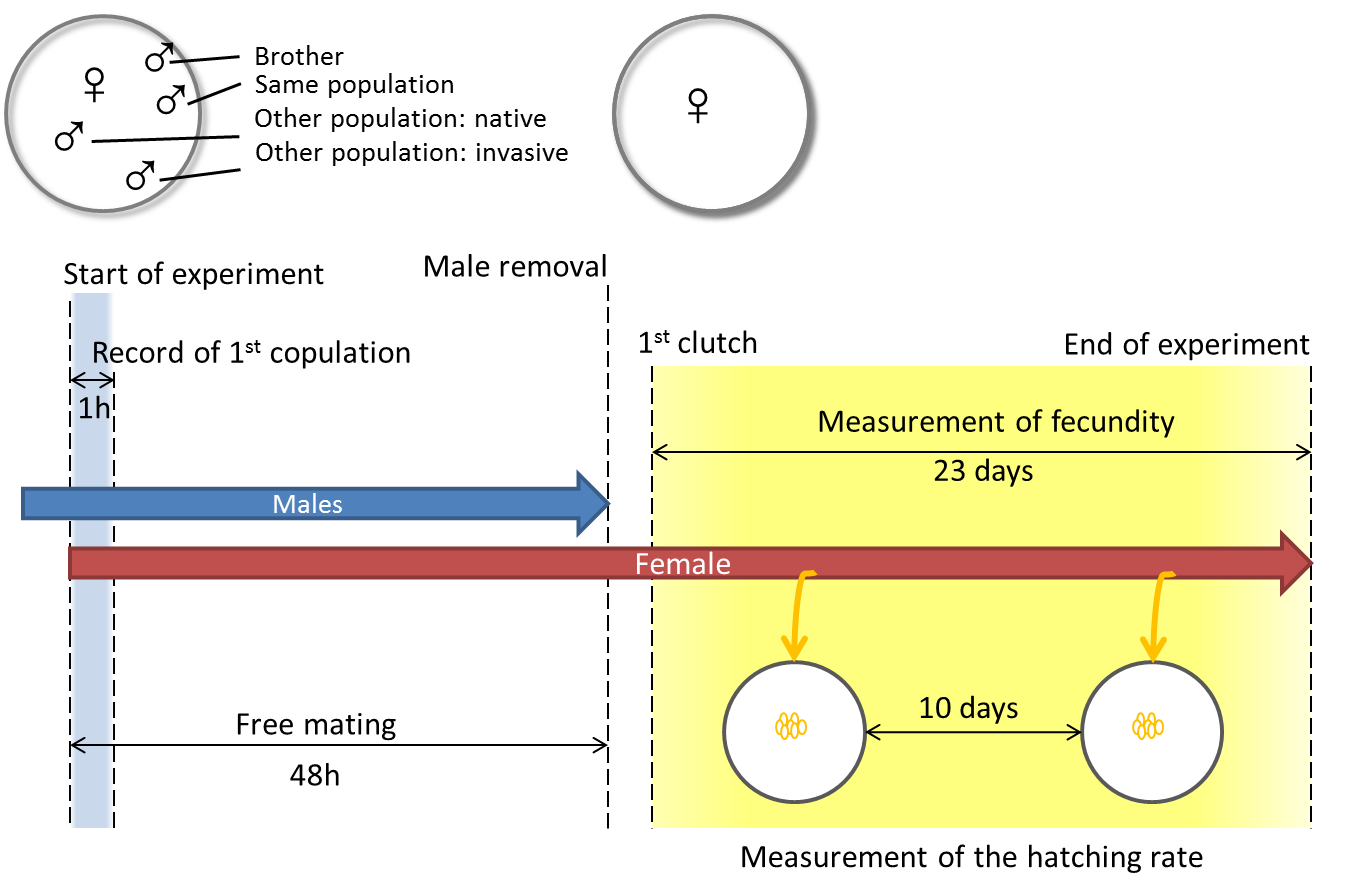

Supplement: Figure S1 — Design of the mate choice experiment. Each female was placed in a Petri dish containing four males: a full sibling, another unrelated male from the same population and two males from other populations, one native and one invasive. The identity of the first male to mount the female was recorded during the first hour and the insects were then left to copulate freely for the next 47 hours. The males were then removed and the females were left alone for 23 days after the laying of the first clutch of eggs. During this period, female fecundity was recorded and the hatching rate of the eggs was estimated from at least one early and one late clutch. The paternity of eight second-instar larvae was assessed by molecular analysis, in two clutches laid at least 10 days apart. (TIFF) [file pone.0077083.s002.tiff]

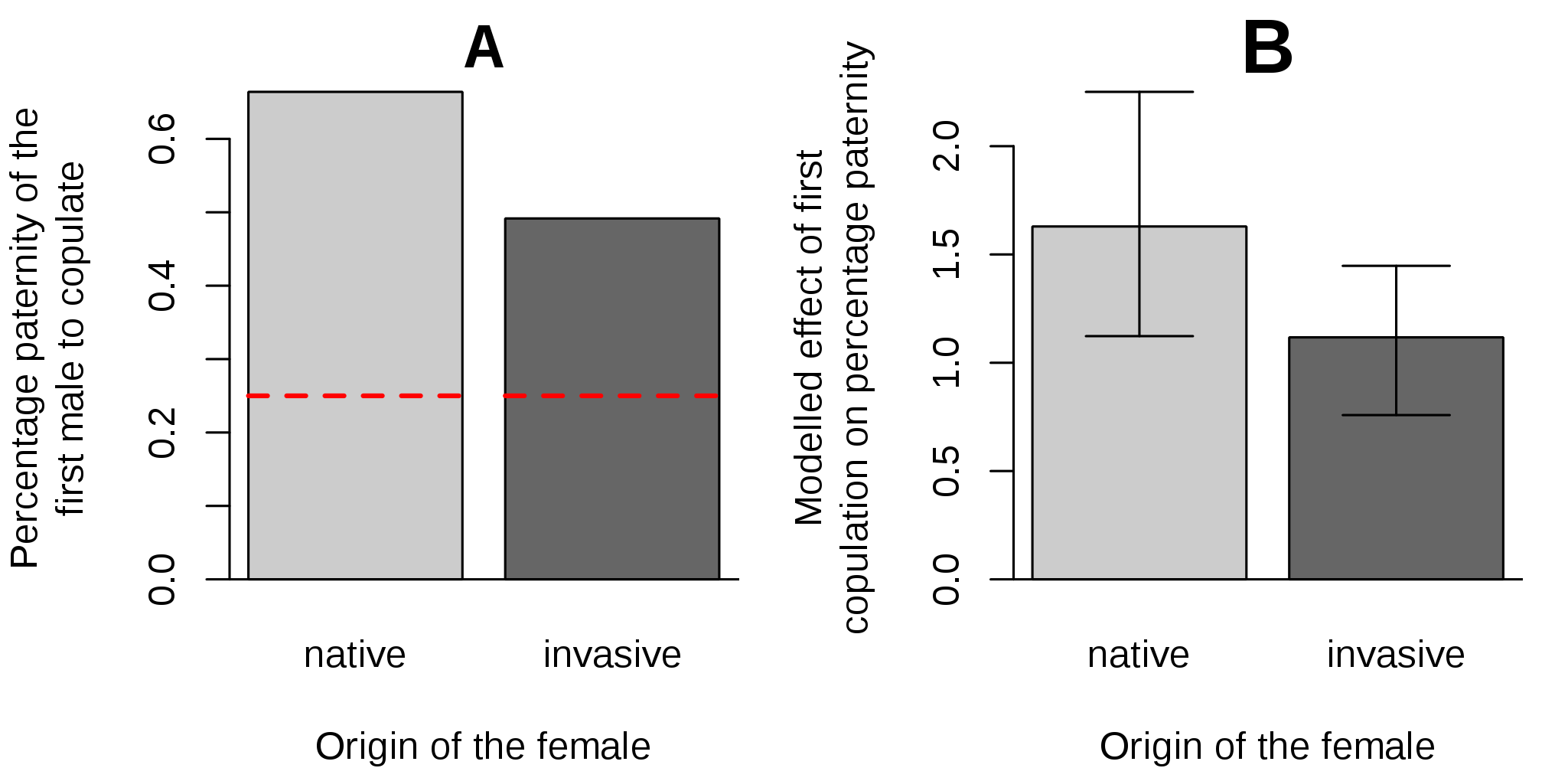

Supplement: Figure S2 — Effect of being the first male to copulate with a given female on percentage of paternity within the female's offspring. A: Observed percentage paternity of the first male to copulate with native and invasive females. Red dashed lines are the values expected under the null hypothesis. B: Model estimates of the effect of being the first male to copulate with a female on percentage paternity, with corresponding 95% confidence intervals. The expected effect with the null model is zero for both native and invasive females. The effect is significant in both cases, but the difference between native and invasive females is not significant (see models P1 and P2 in Tables 2 and 4). (TIFF) [file pone.0077083.s003.tiff]
