# Supplementary material for: Increase in Male Reproductive Success and Female Reproductive Investment in Invasive Populations of the Harlequin Ladybird Harmonia axyridis
Source: PLoS One. 2013 Oct 18;8(10):e77083. doi: 10.1371/journal.pone.0077083 (PMC3799855; doi:10.1371/journal.pone.0077083)
Supplement: Appendix S1 — Details of the statistical model of competition. (DOCX) [file pone.0077083.s005.docx]

Increase in male reproductive success and female reproductive investment in invasive populations of the harlequin ladybird, *Harmonia axyridis*

Short title: Reproductive success in invasive populations

Guillaume J. M. Laugier †*, Gilles Le Moguédec §, Ashraf Tayeh †, Anne Loiseau †, Naoya Osawa ‡, Arnaud Estoup † and Benoît Facon †.

† Inra, CBGP, UMR 1062, Montpellier, France

§ Inra, AMAP, UMR 0931, Montpellier, France

‡ Laboratory of Forest Ecology, Graduate School of Agriculture, Kyoto University, Kyoto, Japan

* Corresponding Author: Guillaume Laugier, Centre de Biologie pour la Gestion des Populations (CBGP), Campus International de Baillarguet, CS 30016, 34988 Montferrier-sur-Lez cedex, France. Tel: +33 4 99 62 33 25 ; Fax : +33 4 99 62 33 45 ; e-mail : guillaume.laugier@supagro.inra.fr

# Supporting information

## ****Statistical modelling of data for male reproductive success****

According to our experimental design, the outcome of male sexual competition is independent between two Petri dishes but not within each Petri dish. This feature precludes data analysis with classic generalised linear models for male traits. We therefore had to construct appropriate models for correct quantification of the potential effects on male success and assessment of complex interaction effects.

### Score for the male

We considered competition between the males to be like a race. In each round, four males ‘run to the finish line’ (mounting the female), so the probability $p_{i,k}$ of a male $k$ winning a race in ‘arena’ $i$ (a given Petri dish) depends on its 'fitness score' $y_{i, k}$ relative to the other three males.

This probability can be written

$$p_{i,k}=\frac{y_{i, k}}{\sum_{k^{'}=1}^{K} y_{i,k^{'}}}, K=4; y_{i, k}\geq0 for all i, k;and \sum_{k} p_{i, k}=1, for all i$$

where the score $y_{i, k}$ can be generally written as

$$y_{i, k}=\exp\left( \sum_{t=1}^{T} \mu_{t_{i, k}} \right)$$

Where $\mu_{t_{i, k}}$ is the effect $t_{i, k}$ (including interaction factors) of male $k$ in arena $i$. If *K*=2, this model corresponds to the classical logistic model.

### Likelihood of the model

More generally, we assumed that a total of *n* independent races took place in each arena. Let $z_{i,k}$ be the number of victories for male $(i,k)$. $Z_{i,k}$ follows a multinomial distribution. The likelihood $L_{i}$ of the results for arena $i$ is the expression of the multinomial distribution:

$$L_{i}=\mathbb{P}\left[ \bigcap_{k=1}^{K} \left( Z_{i, k}=z_{ik} \right) \right]$$

$$=\frac{\left( \sum_{k=1}^{K} z_{ik} \right)!}{\prod_{k=1}^{K} (z_{ik})!}\prod_{k=1}^{K} {p_{ik}}^{z_{ik}}$$

As only one race per arena was considered, we have, for all *i* and all *k,* $z_{ik}=0$ or $z_{ik}=1$, thus $z_{ik}!=1$ in all cases. The likelihood expression can thus be simplified to

$$L_{i}=\prod_{k=1}^{K} {p_{ik}}^{z_{ik}}$$

We can now obtain an expression of the likelihood $L$ of the data over all arenas. As the events in one arena are independent of those in any other arena, this expression is simply the product of the likelihood of the results of each arena:

$$L=\prod_{i=1}^{I} \prod_{k=1}^{K} {p_{ik}}^{z_{ik}}$$

The log likelihood of the model is thus

$$\log L=\sum_{i=1}^{I} \sum_{k=1}^{K} z_{ik}log(p_{ik})$$

### ****Model parameter estimations and statistical tests****

**Model fitting was achieved by maximising the previous log-likelihood. The confidence intervals of the parameters were estimated from 2,000 bootstrap samplings. Models were compared using**
